# Gender-Related Beliefs, Norms, and the Link With Green Consumption

**DOI:** 10.3389/fpsyg.2021.710239

**Published:** 2021-12-03

**Authors:** Ziyue Zhao, Yuanchao Gong, Yang Li, Linxiu Zhang, Yan Sun

**Affiliations:** ^1^Key Laboratory of Behavioral Science, Institute of Psychology, Chinese Academy of Sciences, Beijing, China; ^2^Department of Psychology, University of Washington, Seattle, WA, United States; ^3^Department of Psychology, University of Chinese Academy of Sciences, Beijing, China; ^4^School of Business, Beijing Technology and Business University, Beijing, China; ^5^Key Laboratory of Ecosystem Network Observation and Modeling, Institute of Geographic Sciences and Natural Resources Research, Chinese Academy of Sciences, Beijing, China; ^6^The United Nations Environment Programme - International Ecosystem Management Partnership, Beijing, China

**Keywords:** green consumption, gender difference, value behavior norm, environment, sustainable, pro-environmental behavior

## Abstract

Although extant literature provided abundant evidence that men and women are different in their environmental behaviors, there is a lack of integration of gender differences in green consumption and the underlying mechanism that associates with these disparities. Therefore, to solve this existing gap, the current paper reviewed existing literature on green consumption with threefold purposes. First, presenting an integrated view of gender-different green consumption patterns along with the relationship of gender-related beliefs and individuals’ pro-environmental behavior based on existing evidence. Second, interpreting how gender differences are generated based on the value-belief-norm (VBN) theory, and the theory of social roles. Third, analyzing previous studies, providing implications for future research, and then proposing suggestions for marketing practitioners in the green products industry. Accordingly, this article compared men’s and women’s different behavior in green consumption and discussed how and why they behave differently. Generally, women show a more positive green consumption intention, consume less carbon, and purchase green products more frequently. Whereas men are doing better than women in terms of environmental knowledge, and in some regions, they express higher concerns about environmental problems. It interprets individual differences in green consumption based on VBN theory from a unique insight—gender. It also identified some barriers for both men and women to participate in green consumption, and then proposed several suggestions to improve the public willingness of engaging in green consumption.

## Introduction

Green consumption is characterized by purchasing green products that are recyclable, renewable, and beneficial to the environment, or minimizes the negative effect on the environment and natural resources during the products’ manufacturing process (McEachern and McClean, 2002; Mostafa, 2007). Unlike regular products, green products, such as biodegradable plastic containers, energy-saving light bulbs, electric cars, and natural perfumes, are less polluting to the environment. Currently, the environmental pollution and natural damage related to humans’ daily consumption has become much more apparent than before (Choi et al., 2015; Han, 2015), which increases the urgency of saving natural resources and reducing the pollutions and greenhouse emission in daily consumption to improve the environment quality. Because of this, converting public consumption into a more eco-friendly way has been considered an engageable and impactive method to reduce the artificial harm to nature, and both policymakers and researchers have been constantly making efforts to promote green consumption during the past few decades (Leonidou and Leonidou, 2011; Cholette et al., 2013). For example, to reduce the carbon emission from transportation and promote the operation of eco-friendly vehicles in the general public, many countries such as Japan, China, Belgium, and other European countries have already provided various subside methods for increasing electric vehicles sales such as bonuses and different levels’ tax reduction for the consumption of incenting the electric vehicles (Ahman, 2006; Han et al., 2011; Hockenos, 2011). Other than promoting green products, researchers also devoted themselves to minimizing environmental costs in the market. As recently, Yavari and Ajalli proposed an effective strategy to decrease companies’ total environmental costs by building up a more environmentally friendly supply chain and reducing carbon dioxide emission in the process of materials distribution (Yavari and Ajalli, 2021). In the investigation of public green consumption intention and behavior, most studies have suggested that women are more supportive of green consumption, whereas there are also arguments supporting that men perform better in some aspects of environmental behavior, such as environmental knowledge, environmental intention, etc. (Diamantopoulos et al., 2003; Pagiaslis and Krontalis, 2014; Rahim et al., 2017), which raises an interesting topic, gender difference.

However, most previous studies that explore the gender differences in green consumption admitted and emphasized the impact of gender-associated social stigma and norms on green consumption (Mohai, 1997; McCright and Sundström, 2013; Swim et al., 2020). For example, connecting green consumption with feminine behavior is an obstacle for men to engage in green consumption because they prefer to perform in a way that aligns with their masculine gender identity, and stay away from feminine characterized activities (Brough et al., 2016; Swim et al., 2020). Therefore, we believe that gender is a meaningful and interesting topic, but gender itself might not be the determining factor that induces distinct green consumption behavior of men and women. Instead, the effect of gender-related beliefs and norms on an individuals’ green consumption is an essential part that deserves a deeper exploration in the entire pro-environmental field. An individuals’ personal value is shaped by the longitude process of socialization and affected by the social stereotype of their own social identity. Since there are different traits of both men and women that society prefers, gender-different personal value has been formed (Di Dio et al., 1996). The personal value affects an individuals’ attitude and willingness to engage in a particular behavior, such as higher altruism making individuals more likely to worry about the natural environment and would like to support activities involved with environmental protection, but egoistic personal orientation restricting an individual’s engagement on green consumption (Blocker and Eckberg, 1997; Bogner and Wiseman, 2004).

Accordingly, men’s and women’s different personal values contribute to their attitude and motivation to green consumption. The attitude to green consumption is also greatly affected by social norms (Mohai, 1997; Stern, 2000; Frantz and Mayer, 2009; Alibeli and White, 2011; McCright and Sundström, 2013). Since based on social norms, women usually take more responsibility for caring for others, and play the altruistic and cooperative role, whereas, the social expectation on men is inclined to more aggressive characters (Alibeli and White, 2011; Gysbers et al., 2014). The green consumption behavior has a strong link with femininity and is represented by feminine characters, and behavior such as saving energy, reducing carbon consumption is less related to the masculine characters such as competence and ambition (Brough et al., 2016). Therefore, compared with women, men usually hold a more avoidable, instead of active attitude to green consumption behavior (Nanggong and Bandu, 2018; Swim et al., 2020). Besides, it is also necessary to clarify the definition of “gender” in this article. Unlike sex, an inborn, unchanged personal identity from a biological perspective, by saying gender, we refer to the identity that was determined by each person and varies based on an individuals’ own social preferences. Also, as a social construct, gender involves or represents various social norms, beliefs, and behaviors that were considered appropriate by the public society for men and women (World Health Organization, 2002). This review aims to integrate previous studies’ results and analyze the links between gender-related beliefs, social norms, and environmental behavior, manifested as green consumption. The necessities and wishes of this review are presented as follows.

First, the exploration of gender differences provides a unique perspective for explaining the individual differences in green consumption behavior. For now, most researchers have discussed green consumption from moral identity, psychological ownership, consumer attitude, and other related factors (Biswas and Roy, 2015; Wang et al., 2019), but overlooked the impact of demographic factors such as gender, age, region, etc. Robert once suggested that studies failed to consider the demographic factors are incomplete (Roberts, 1996). Gender, as suggested by Baker and Ozaki, is unignorable not only because it was an important reason behind these psychological differences affecting green consumption but also since that gender equality and different social stigma toward men and women have a huge effect on their green consumption and other related pro-environmental behavior (Pickett-Baker and Ozaki, 2008).

Second, by exploring the effects of gender-related beliefs and norms on green consumption behavior, we can analyze the popular theories that are used to explain pro-environmental behavior from one more perspective, gender. The Value-Belief-Norm (VBN) theory proposed by Stern is an authoritative theory used to interpret the effects of personal values on individuals’ behavior under particular social circumstances. It emphasizes the critical roles of personal values, beliefs, and norms in stimulating individuals’ pro-environmental behavior, as based on VBN theory, prosocial behaviors are more likely to happen when personal values, beliefs, and norms are activated together. The VBN theory is frequently used to explore individuals’ differences in various social behavior. Egoistic, altruistic, and biospheric value orientations that previous studies typically use to discuss distinct environmental behavior also align with the belief in the VBN theory (Dietz et al., 2002; De Groot and Steg, 2007; Xu et al., 2021). Besides, the theory of green purchase behavior that was established recently admits the role of the VBN theory in exploring green consumption behavior as well (Han, 2020). In the national survey conducted by Stern in 1994 toward 420 samples, among many of the environmental theories, the VBN theory provides a more precise prediction of individuals’ support for environmental behavior (Stern et al., 1999). Accordingly, the current paper selects the VBN theory as the theoretical basement and suggests that it is also adaptive for interpreting the distinct gender-related beliefs and norms in green consumption behavior. After further exploration in this field, it will be possible for researchers to examine whether other popular theories can be explained from the gender dimension.

Third, even though researchers verified that men’s and women’s pro-environmental behavior are usually different, there are still some arguments and opposite findings in existing studies. Most studies claim that women are more eco-friendly than men since they engage in more pro-environmental behavior, and express stronger environmental concern (Krauss, 1993; Zelezny et al., 2000; Vainio and Paloniemi, 2014), but there are studies arguing that men’s environmental attitude is more positive than women, and they also have more solid environmental knowledge regarding the comprehension about environmental information and issues (Banerjee and McKeage, 1994; Mostafa, 2007; Ping and Linxiao, 2020). Therefore, whether men or women are more eco-friendly has not come up with a conclusion yet. This review aims to provide an objective summary and discussion based on previous findings regarding the link between gender-associated beliefs and pro-environmental behavior, especially green consumption and related environmental intention.

Fourth, the current review wishes to provide guidance for green businesses on how to make effective publicization targeting various consumers, especially distinct marketing strategies to men and women through analysis of relevant studies about the relationship between gender and green consumption. Since men and women perceive the same advertisement or interpret the same information differently and their motivations of purchasing green projects also have discrepancies, merchants need to be aware of the gender differences regarding green products’ preference when they design, produce and publicize products (Brough et al., 2016; Nanggong and Bandu, 2018).

In conclusion, this article not only summarizes the existing literature that discusses gender different behavior, intention, and attitude in green consumption but also integrates the social and personal triggers of this difference. It is novel to examine gender differences in green consumption from the perspective of the VBN theory and analyze the connection between gender-related social norms and green consumption behavior. Additionally, by comparing men’s and women’s behavior and exploring the reason behind this difference, this article identified several factors that are potentially disadvantageous to green consumption, and then proposed suggestions that specifically target these obstacles to the green products industry. The first part of this review synthesizes specific gender differences in green consumption behaviors and related aspects such as green consumption attitudes, environmental knowledge, etc. Next, we explain the distinct gender beliefs and norms from the VBN theory and then analyze the social mechanism behind this discrepancy in green consumption behavior. In addition to the brief comparison of green consumption between men and women, the current article focuses on discussing exactly how gender-related beliefs and norms play a role in green consumption. Besides, by analyzing the inconsistent findings in this field, we indicate potential factors that interact with gender to affect green consumption together. In the end, after a deep exploration of the association between gender-related beliefs and green consumption, we analyze men’s and women’s different preferences toward green marketing and provide some practical suggestions for future researchers and marketers.

## Methodology

Multiple databases were used in the process of searching qualified articles for this literature review. Google scholar was the initial database that we used for getting an overview of the currently available literature, and then through the University of Washington Library search database selectors, we chose Science Direct, PsycInfo, Elton B. Stephens CO (EBSCO), and Taylor and Francis as our three primary databases, and part of the articles are searched from SAGE, CNKI, PubMed, SpringerLink, and JSTOR.

The terms used for searching literature include: *Pro-environmental Behavior; Environmental Behavior; Environmental Attitude; Environmental concerns; Environmental Knowledge; Environmentalism; Personal Value; Altruism; Eco-friendly Intentions; Green food; Green consumption; Green Marketing; Gender Differences; Gender identity; Green consumption; Green consumers; Social stereotype*. These words are combined in various ways with the AND as the connection. Most of these terms are generated from keywords of articles searched in Google Scholar using the initial term “*gender differences in green consumption*.” Besides direct searching using keywords on the database, a part of the articles was selected through the Snowball method by identifying an article as a start point and selecting other articles derived from it, but each article includes at least one or more search terms that we indicated earlier.

The inclusion criteria for the selections are described as follows. First, the questions that these studies examined should correlate with the topic that the current review focuses on. Second, the articles have to be either from peer-reviewed journals or book chapters, except certain data and definitions are selected from the WHO. Third, the articles should be published within the year 1990 to 2021, and the data analyzed in each study should also be later than 1990, any articles published earlier than 1990 will be excluded. After considering these factors, 96 articles were selected and used as references for this current review.

## Comparison of Green Consumption Between Men and Women

### Green Consumption Intention

Gender differences in green consumption are significant with women expressing a stronger green consumption intention and purchasing more eco-friendly products than men (Banerjee and McKeage, 1994; Rahim et al., 2017). On a daily basis, women are more inclined to use household green products such as eco-labeled products (Loureiro et al., 2002; Vermeir and Verbeke, 2006; Han et al., 2011), with a stronger willingness to accept ecological advertisement than men (Yu, 2020). It has been found that men not only show a lower acceptance of the benefits of green products, they are also more likely to suspect the effectiveness and ecological benefits of these products (Chekima et al., 2016). Men usually determine whether to purchase green products based on the function, utilization, and personal responsibility to nature (Tung et al., 2017). On the other hand, the primary drives of women’s green consumption are the products’ ecological impacts, instead of whether they are practical or not. Tung et al. (2017) indicated that except for environmental concerns, there are more factors promoting women’s green consumption than men. For example, women might purchase eco-friendly apparel because its fabric looks good, or might express an obvious supportive attitude to green products due to the wish of strengthening their self-identity (Tung et al., 2017). Gender differences in green consumption do not only exist in adults but also appear at young ages with adolescent girls showing a stronger green consumption intention. Also, compared with boys, adolescent girls are more likely to be affected by peers on green consumption, which promotes them to perform even better (Han et al., 2011).

### Carbon Consumption

Most previous researchers have proven that men usually consume more carbon than women, which is closely related to their traveling ways, eating habits, and substances usage, such as tobacco or alcohol (Räty and Carlsson-Kanyama, 2010; Waygood and Avineri, 2016; Liu et al., 2019). Studies conducted by Loureiro as well as other researchers within the US and many European countries’ adults show that women tend to eat more vegetables or fruits, and spend more money on purchasing organic, seasonal, or chemical-free food (Loureiro et al., 2002; Vermeir and Verbeke, 2006). Women also waste less food than men, which might have a relationship with their frequent purchasing of organic food since some research suggests that individuals who buy more organic food tend to waste less (Barr, 2007; Principato et al., 2015). Whereas men’s purchasing rate of meat, protein-rich products, extra-processed food, beverage, and other similar drinks is much higher than women, and men also report a higher frequency of eating at restaurants and using tobacco (Elmståhl et al., 1999; Biloukha and Utermohlen, 2000; Fraser et al., 2000; Liebman et al., 2003). Men’s strong preference for protein-rich diets has a significant potential risk of increasing energy consumption and gas emission that contribute to global warming and the greenhouse effect (Carlsson-Kanyama et al., 2003).

Another aspect leading to men’s higher energy consumption is their driving frequency. Generally, traveling through public transportation is more attractive to women, that is, they take public transportation such as trains, links, buses, or taxis more often than men (Marian, 2020), and women own fewer cars than men (Bernard et al., 1997; Linderhof et al., 2001; Sánchez and González, 2016). The gender-different travel pattern is affected by multiple factors such as access to cars, vehicle reliance, environmental norms, gender stereotypes, trip length, and the number of children, etc. For most countries, women have less access to private cars than men (Matthies et al., 2002; Colin et al., 2004), which limited their usage of private vehicles. However, even though the number of women who own cars or get driver’s licenses increased, they are not as dependent on cars as most men have. For men who have been drivers for decades, they have developed a reliance on cars, which decreases their willingness to take public transportation even more (Matthies et al., 2002; Claudia and Barbara, 2004). Moreover, Matthies has demonstrated that since women are more concerned about environmental conditions and tend to have more safety or health concerns as well, they are more active in environmentally friendly behavior in their life, such as taking public transportation (Matthies et al., 2002). Whereas, women’s driving frequency is higher than the average in some conditions. Since most women are taking responsibility for taking care of their children and family trivia, the number of children is a factor that might affect this common gender-different travel pattern. When examining how nursing children or taking care of family is associated with gender differences in transportation patterns, a concept called “trip chaining” was been used by researchers. The word “trip” always refers to a process that individuals go through from their start point to their destination. Similarly, trip chaining means when there are many stops between an individual’s start point and their final destination (Nancy and Yukiko, 2004). In this article, we primarily focus on the stops within people’s daily commute, and how that suggests gender differences. In the 2001 National Household Travel Survey, 4.3 million women drop their children off or pick them up on their way between their house and workplace, but only 2.7 million men are responsible for this. Also, most women’s trip chaining is because of purchasing household stuff or dealing with family works, especially in the family having multiple children (Nancy and Yukiko, 2004). Self-driving is much more convenient for women to do these relevant jobs, and therefore gender differences in the frequency of taking transportation are less relevant in women who have more than one child (Strathman and Dueker, 1995). Gender different driving frequency and attitude to taking public transportation does not only exist in adults but as Matthies suggested, driving has been ingrained in men’s minds since their childhood. Most boys have been hoping to drive at the age of 10, which contributes to their future usage of vehicles, instead of taking public transportation (Matthies et al., 2002). Other than driving frequency, Delhomme’s investigation indicates that there is also a gender difference in driving habits, as women engage in more eco-friendly driving behavior such as using the accelerator in a more conversive way or controlling the driving speed to be as stable as possible (Delhomme et al., 2013). These traveling differences also include hotel choices, since compared with men, women travelers would like to pay a higher price to choose green hotels that enact eco-friendly managing ways and purchasing procedures, and they also are more willing to recommend these green hotels to others (Han et al., 2011). However, while men usually consume much more carbon relative to women, they do not feel as much guilty as women for their non-eco-friendly life (Räty and Carlsson-Kanyama, 2010).

### Different Opinions

In contrast with the opinions supporting women’s better performance on green consumption, some results suggest that men perform greater than women in some aspects. For example, in investigations about daily commuting, Heesch et al. (2012) indicate that compared with women, men are more likely to cycle to work or to school.

Pagiaslis and Krontalis (2014) also suggest that men are more knowledgeable in renewable energy, especially biofuels, and show a stronger purchasing intention of biofuels than women. Also, there are studies that argue that men show a stronger attitude toward sustainability, which encourages them to use less polluted products. Considering the consistency of individuals’ environmental attitudes and their actual green consumption behavior, Banerjee and McKeage (1994) and Mostafa (2007) suggest that the linkage between environmental attitude and green consumption is significantly stronger in men than in women. Additionally, plenty of studies report that the performance of men in objective environmental knowledge is higher than women, and know more about various types of environmental problems (Schahn and Holzer, 1990; Diamantopoulos et al., 2003). The comparison of gender different behavior in green consumption, as well as the inconsistent findings, are all summarized in Table 1.

**TABLE 1:** Comparison of gender differences in green consumption behavior and intention.

**Sources**	**Subjects**	**Thesis**	**Findings**
Banerjee and McKeage (1994)	Students from the Northeastern University	The relationship between environmentalism and materialism	Environmentalism negatively correlates with materialism, and women show a significantly higher environmentalism and value environmental significance much more than men in consumer behavior
Barr (2007)	U.K. residents	The effect of environmental attitude on household waste management	Women are more likely to intentionally reduce their waste than men
Biloukha and Utermohlen (2000)	Ukrainian citizen	Examine food perception and consumption across Ukrainian citizen	Men perceive food as less costly than women and less likely to value food
Blocker and Eckberg (1997)	U.S. general public	Explore the reason of why women are more concerns about environmental related issues than men from the social perspective	Women shows more personal concern to environment than men, also women who are more knowledgable or in a higher social status engage more environmental behavior. Women’s caregiver role also positively affect their environmental behavior and concern
Brough et al. (2016)	American, U.K, and Chinese adults	Whether the stereotype of gender identity affect men’s desire of conducting green behavior	The association between feminine and green products, green behavior increases men’s concern about their masculine identity, which then drop their willingness of engaging green behavior.
Carlsson-Kanyama et al. (2003)	Swedish publics	Examine the pattern and result of daily food taken and energy input in Swedish population	Women consume more vegetables and fruits instead of meats than men
Chekima et al. (2016)	General public in Malaysia	Investigating effects of demographic factors on green consumption	Gender has significant effects on green consumption behavior where women are more trust to eco-label and purchase more green products
Delhomme et al. (2013)	French drivers	Examining the frequency of ecofriendly driving behavior	Women have more eco-friendly driving behavior
Diamantopoulos et al. (2003)	UK public	Whether there is an association between socio-demographics and green consumption	Women have a stronger attitude toward environmental quality, show “greener shopping habits,” and are more active at recycling. Whereas no gender difference shown on environmental knowledge.
Ping and Linxiao (2020)	Chinese adults	The relationship between gender and proenvironmental behavior in China	Chinese women are more active in private pro-environmental behavior, whereas men involves more public pro-environmental behavior. Compared to men, women have a weaker environmental problem perception.
Han et al. (2011)	U.S. hotel consumers	How does customers’ intention of visiting green hotel differ across gender and other social demographics?	Women think eco-friendly intention as more favorably than men, have a higher intention to purchasing ecofriendly products, and show more eco-friendly behavior
Han (2020)	U.S. hotel consumers	The association between customers’ eco-friendly attitude and their intention to visit “green hotel,” and exploring whether this intentions differ across gender	Eco-friendly attitude have a positive relationship with green purchasing intention. Women holds a more positive attitude toward green consumption and shows stronger desire to choose the green hotel
Heesch et al. (2012)	Adults from Queensland, Australia	Women’ and men’s recreational and transport cycling pattern	Men are more likely to ride bicycle in daily commute than women
Schahn and Holzer (1990)	German adults	Explore gender difference and environmental knowledge and attitude	Women are more environmentally concerned about household issue, but men know more environmental knowledge
Krauss (1993)	White, African American and Native American women working class	Examine women’s attitude toward protests of toxic waste issues	Women’s social identity, especially the mother role, closely relates with their motivation for supporting the toxic waste related protest.
Liebman et al. (2003)	Adults from rural community in six rural communities in Wyoming, Montana, and Idaho	Assess the gender different health eating pattern	Women are more likely to eat vegetables, fruits, and other fiber rich food than men. Also, they tend to prefer to eat at home rather than going out
Liu et al. (2019)	Chinese college	The low-carbon consumption intention and behavior in Chinese college students	Women have a higher intention on purchasing low-carbon products than men
Loureiro et al. (2002)	U.S. consumers	Investigating consumers’ willingness to buy eco-labeled fruits	Women are more likely to buy ecolabeled fruits than men
Marian (2020)	General public from Auckland, Dublin, Hanoi, Helsinki, Jakarta, Kuala Lumpur, Lisbon, and Manila	Examining gender different transporting mode in eight different countries	Women prefer public transportation over driving than men.
McCright and Sundström (2013)	Swedish general public	The relationship between gender and environmental concern	Women show greater environmental concern than men, especially to environmental problems. This difference is associated with women social rules
Mohai (1997)	U.S. college students	Explore gender difference in concern and behavior of environmental related issues, and examine the explanation behind this difference	Women have a better performance and greater concern on resource conservation, nature preservation, pollution, global environmental problems, and neighborhood environmental problems. This difference may related with their social roles and identity. The interaction between race and gender also exist
Mostafa (2007)	University students in Egypt	The relationship between gender and environmental knowledge, perception, and green purchase attitude	Men are more concern to environmental and show more intention to purchase green product
Nanggong and Bandu (2018)	Indonesia public consumers	Investigating gender differences in environmental attitude and sustainable consumption in the use of paperless technology	Men tend to perceive more benefits of adopting paperless technology (etickets), whereas women are more likely to use paperless technology out of the concern for environment.
Ozanne et al. (1999)	U.S. homeowners	Explore gender difference in environmentalism	Women concern environment more than men, prefer eco-friendly and ecological certificated products. Women more active in household environmental behavior, but less likely to participate public environmental behavior than men
Pagiaslis and Krontalis (2014)	U.S. general public	The linkage between environmental concern and green consumption behavior, and the individual differences based on demographics	Environmental concern positively related to green consumption intention, and gender difference exists by showing that women are more concerned to environment but men show a higher intention of purchase reusable energy.
Rahim et al. (2017)	Facebook and Twitter adult users	Investigate effects of demographic factors on consumers’ green product purchase intention	Women have a significantly higher green product purchasing intention than men
Räty and Carlsson-Kanyama (2010)	Data obtained from Swedish version of the Energy Analysis Program	Total energy use and gender different energy consumption pattern in European countries	Men consume more energy than women, especially in travel, eating outside, food intake pattern, alcohol and tobacco usage
Muralidharan and Sheehan (2018)	England general public	Examine gender differences in guilt perception and whether guilt related to reusable shopping bag usage	Economic concern is an obvious motivation for women to bring or use reusable shopping bags
Swim et al. (2020)	U.S. undergraduates	Social consequences of engaging gendered environmental behaviors	Both men and women prefer to engage activities that are consistent with their gender identity. Most of proenvironmental behavior are more feminine, which makes men are not likely to engage those behavior.
Tung et al. (2017)	General Public in the United States	Understanding gender different attitude to green consumption and the motivation behind their purchase of green products	Motivations of men and women’s purchase behavior are different and women express more positive attitude to green consumption.
Vainio and Paloniemi (2014)	Adult in Nordic Countries	Examine whether the attitude toward science has effects on proenvironmental consumption	Positive attitude toward science increases the likelihood of ignoring the importance of proenvironmental behavior. The confidence that science decreases the necessity of pro-environmental behavior directly increases with pro-environmental consumption.
Waygood and Avineri (2016)	Adults from Brazil, China, Great Britain, Italy, and Spain	Gender difference in environmental knowledge and concern, and their different behavioral response to the climate change	Women has stronger and more positive behavioral reaction in their transportation way facing the climate change
Xiao and Hong (2017)	China general public	An update study of men and women’s environmental knowledge and attitude	Different from the study of 7 years ago, women’s environmental knowledge is still slightly lower than men, but the environmental attitude of men and women are almost equal
Xiao and Hong (2010)	Chinese general public	Examine existed gender difference in environmental knowledge, attitude, and concern in China	Women engage more household environmental behavior. Men show greater environmental concern as well as knowledge than women
Yu (2020)	General public	Examining effects of gender on the acceptance of green advertisement	Women are more easily to accept and how a more positive attitude than men toward green advertisement. In contrast, men tend to be more doubtful for green advertisement.
Zelezny et al. (2000)	Primary and secondary school students in California; Undergraduates from Europe, Latin America, and Unite States who speak English and Spanish	Whether the gender difference of environmentalism across-countries and whether it also exists in children	Gender related differences of environmentalism is an across countries issue, and also exists in children. Women’s stronger environmentalism associates is possibly because of the socialization process.

### Factors to Explain the Inconsistent Findings

In the discussion of gender differences in green consumption, several potential factors have been identified for explaining the inconsistent results. Environmental attitude, which refers to an individuals’ general emotions toward environmental protection and damages (Pe’er et al., 2007), has been frequently mentioned by previous studies as one of the strong predictors of consumers’ green consumption and their choices between common products and green products (Abdul-Muhmin, 2007; Jansson et al., 2010). Consumers who hold a more friendly pro-environmental attitude usually engage in green consumption behavior more frequently, and for example, they report a higher likelihood to purchase electric vehicles instead of gasoline cars (Mostafa, 2007). Besides environmental attitude, environmental knowledge is also identified as a predictor of green consumption behavior because it has a critical effect on an individuals’ green consumption and contributes to the establishment of personal norms favoring environmental protection and green consumption, and individuals who hold a deeper environmental knowledge would like to spend more money for eco-friendly products (Vining and Ebreo, 1990). Also, Xiao and Hong reported that men have more environmental knowledge and show greater environmental concern than women based on their investigation of the Chinese general public (Xiao and Hong, 2010), which then supports the positive relationship between environmental knowledge and environmental concern. However, in their follow-up study in 2017, they found that even though women’s environmental knowledge was still lower than men’s, their environmental concern is about at the same level as men’s (Xiao and Hong, 2017). In Xiao’s opinion, one factor accounting for the distinct result of these two experiments is education level. In his prior study, subjects were chosen from the general public in 2003 when men’s average education years was significantly higher than women. Whereas, in the study in 2017, Xiao claimed that the educational discrepancy between men and women is appreciably smaller than before and even does not exist in populations under 30 years old. Zeng et al. (2014) also suggested that the gap between men’s and women’s education levels is consistently shrinking, and there is almost no difference in the educational attainment of men and women. Inequity has almost disappeared in the public chances of receiving primary and middle school education either, and what is noticeable is that in some urban areas, girls are likely to have more educational chances and get a higher average academic performance than boys (Zeng et al., 2014). Therefore, this disconnection that occurred in Xiao’s two experiments might be because of women’s increased education level, which contributes to their awareness of environmental behavior even at the relatively lower level of environmental knowledge.

From the previous studies comparing gender different green consumption, we found that inconsistent results are very likely to be generated when studies are conducted in different regions (Mostafa, 2007; Heesch et al., 2012; Ping and Linxiao, 2020). Because of the differences in social circumstances between developed and developing countries, the associated gender belief and social norms are clearly different, such as the seriousness of social stigma to men and women as well as the quality of education as mentioned earlier, which might generate opposite results when shifting the focus from developed countries to underdeveloped countries (Kunovich and Kunovich, 2008; Jones et al., 2014; Pulsipher et al., 2017). When choosing samples from the Arab countries, the result does suggest that men report a higher environmental concern and more positive attitudes toward green consumption, which contradicts with the findings in developed countries (Mostafa, 2007). Alibeli and White (2011) explained this difference from the perspective of opportunities of getting in touch with nature. They claimed that in these countries, the intense and longitude patriarchal social norm requiring women to be responsible for housework restricted their opportunities of connecting with nature and considering environmental problems, which then make them less likely to be aware of the environmental issues and the necessity of protecting the environment (Alibeli and White, 2011). Regional differences related to many social and individual differences might generate opposite results. For instance, women’s environmental attitude is generally more positive than men’s in Western countries, whereas, many studies conducted in China showed that men’s environmental attitude is relatively more friendly than women due to the different levels of environmental knowledge (Xiao and Hong, 2010; Ping and Linxiao, 2020).

## Theoretical Frameworks

As a specific branch of private pro-environmental behavior, green consumption is also considered as pro-social behavior. Mohai, Frantz, and other scholars explained the gender differences from the sociological perspective, which emphasizes the role of social expectation and social norms in shaping individuals’ green consumption, as well as impacts of a particular social context on men’s and women’s pro-environmental behavior (Mohai, 1997; Frantz and Mayer, 2009; McCright and Sundström, 2013; Swim et al., 2020). As we discussed above, even though multiple causes contributing to the gender differences in green purchasing behavior were identified by previous studies, they have not been integrated and analyzed based on the specific social mechanism yet (Nolan and Schultz, 2015). To remedy this gap, we are novel to analyze gender differences from both internal and external dimensions, building on the VBN theory and social expectation separately.

### Analyzing Gender Differences in Green Consumption From the Value-Belief-Norm Theory

The role of Stern’s VBN theory in explaining pro-environmental behavior has been widely accepted, and because the value-belief-norm-theory derives from the norm activation model, we also include some opinions of the norm activation model in our analysis. Purchasing green products can be caused by feelings of obligation to engage the eco-friendly consumption. The VBN theory builds a connection between personal cognition and perception of the environmental problem to the emotional reaction toward particular social circumstances and moral obligation. It also explains how they interact with each other and contribute to the personal norm and then cause individuals’ green consumption behavior (Stern, 2000).

A considerable amount of studies have indicated that personality and the nurturing attitude are positively associated with a higher perception of consequences about environmental changes and stronger responsibility of protecting the environment (Ozanne et al., 1999; Zelezny et al., 2000). The nurturing attitude is a critical part of the social norms and social expectations of women. The feminine social norms have started to affect women since they were young, as girls were expected to purchase plush toys and pretend to play the caring role for their toys, such as building a home for their dolls or covering up stuffed animals for sleep (Gysbers et al., 2014). Girls are also being educated to value others’ needs and take care of family members voluntarily (Betz, 2006). Due to the eagerness of being valued and accepted by society, women’s behavior usually aligns with social expectations, and their personal value is shaped by feminine social norms (Hoffman, 2001; Gilbert and Kearney, 2006). Women’s eco-friendly personal norm is closely related to the nurturing attitude to their children and family, and in turn, encourages their green purchasing behavior (McCright and Sundström, 2013; Brough et al., 2016). The nurturing attitudes toward their children and family members also strengthen their concerns about the environmental problems since environmental quality has a close relationship with their life and family’s health, which lead researchers to indicate that women’s green consumption behavior comes from the concern about their family members (Zelezny et al., 2000; Muralidharan and Sheehan, 2018; Migheli, 2021). Whereas, gender-different caring and protective intentions toward the environment also correspond with men’s and women’s different personal values. Corresponding with women’s social norms, their personal value that is prone to the concerns of others (Mohai, 1997) improves their altruism, which cultivates the formation of their social-altruistic value.

Since green consumptions and other related pro-environmental behavior is a form of prosocial behavior, it was also more prevalent among individuals who hold altruistic personal values (Bogner and Wiseman, 2004). Social altruistic value is characterized by altruism in Stern’s VBN theory, and under this social-altruistic orientation, pro-environmental behavior is motivated by the concern of others. The opposite of social-altruistic orientation is the egoistic orientation where people tend to prioritize their own needs. Men tend to report a higher egoistic orientation, in this case, they usually pay much attention to the benefits that environmental behavior can bring to themselves, and sometimes choose eco-friendly products because of their own needs (Dietz et al., 2002). For example, Nanggong and Bandu (2018) investigation of whether and why people will use electric tickets shows that men usually consider more about the lower price and higher convenience brought by E-tickets, but the benefits to nature such as saving paper and protecting the forest are always the top motivations of women to use E-tickets. In addition to the economic benefit, Griskevicious proposed that satisfying personal psychological needs may also be a reason why men engage in prosocial behavior since compared with women, they can feel much more personal value and self-satisfaction through prosocial behavior. This is partially why men would like to engage in public pro-environmental behavior in front of others (Griskevicius et al., 2010). Moreover, distinct social norms to men and women strongly influence their attitude not only to green consumption behavior but also to others who are involved in green consumption as well. Society tends to associate men with more aggressive characteristics, such as competence or authority, Swim’s study shows that when it comes to supporting climate change policy, men tend to consider supporters who are motivated by protecting the climate as more feminine, but think those who are motivated by contributing to global economic sustainability as more masculine (Swim and Geiger, 2018). Thus, it is unavoidable that men will develop a more egoistic attitude toward green consumption and other pro-environmental behavior under the impact of social norms.

As proposed in Stern’s VBN theory, all the constructs included in these value orientations are devoted to the formation of an individuals’ norms and the consequences that they pay attention to Stern (2000). The value orientation that works most efficiently at encouraging green purchasing behavior is the biospheric orientation. Individuals who report a greater biospheric orientation are more sensitive to the negative consequences of products with higher risks of bringing potential contaminations to nature. Individuals with this value orientation not only engage more pro-environmental behavior but also insist on that for a significantly longer period than others. However, what has the weakest positive connection with environmental concerns and behaviors is egoism, which is more common among men. Even though some researchers argue that women’s green purchasing behavior is also out of the consideration of themselves or their family instead of the biosphere (Muralidharan and Sheehan, 2018), generally, women’s stronger social-altruistic and biospheric orientation with higher altruism make them concern nature for its own sake and take environmental protection as their own obligation, which improves their green purchasing behavior a lot (Bogner and Wiseman, 2004). Additionally, how much individuals value their interpersonal relationship also affect their attitude to the environment. Women take their relationship with others more seriously than men, which affects how they judge their relationship with nature and how much they would like to put themselves into it. Customers who have a closer relationship with nature are more likely to have a stronger feeling of obligation to protect the environment and therefore to purchase green products (Blocker and Eckberg, 1997; Ping and Linxiao, 2020). In contrast, men’s intense egoistic orientation decreases their activism and persistence in green consumption.

### The Effect of Social Expectation on Men’s and Women’s Green Consumption Behavior

Besides the impact of personal values and norms, the external factor, social expectation, also has a significant effect on gender differences in green consumption. The distinct social expectation of men and women comes from the long socialization process and the derived social roles that assign to them since their childhood (Blocker and Eckberg, 1997; McCright and Sundström, 2013). As we have already mentioned, women are socialized to be nurturing to others, and in the family, they are usually assigned the “caring” role, and society expects them to show more motherhood characteristics and consider more welfare of others.

Instead, men’s social roles are more prone to making money, providing the economic foundation, controlling others, and occupying, so under the socialization pressure, men are more willing to conquer nature and utilize the natural resources for the development of the economy rather than considering the needs of the environment (McCright and Sundström, 2013). Besides, the social stereotype of men and women also has a huge impact on their choice of green products. Characteristics such as caring, protective, friendly, concerned, voluntary, and compassionate, were attributed to women, and as mentioned above, behavior that corresponds with these characteristics is labeled as feminine behavior (Krauss, 1993; Zelezny et al., 2000). In contrast, behavior expressing the traits of fighting, proactive, conquering, and leadership has been aligned with masculine characteristics. Under the social gender stereotypes, it is less likely for men to ascribe environmental protection as their own responsibility and then generate related eco-friendly personal norms. From this gender role alignment perspective, green consumption is closely associated with feminine features. As we expected, individuals are all more likely to be gender conformity by conducting the behaviors that align with their gender role (Swim et al., 2020), and the existence of this gender stigma of green consumption keep men from purchasing ecological products even more (McCright and Sundström, 2013). For example, men would not like to choose organic food and use reusable shopping bags to keep themselves from feminine traits (Brough et al., 2016), but women’s social roles bond them together with these behaviors, which even encourage their green consumption. Because of this deep gender social stereotype, the public prefers individuals who conduct the behavior corresponding to their social identity and tend to keep the distance from those who have opposite behaviors or hold misunderstandings to them, which usually refers to the punishment of gender-bending. An interesting finding showed that compared with men, women who refuse to engage in eco-friendly consumption will more likely be avoided by others, and similarly, men who constantly use recyclable shopping bags might be socially distanced or considered as gay (Swim et al., 2020). Thus, from the dimension of distinct social roles and individuals’ desire of conforming the social expectation, it is much easier for women to engage in green consumption and take other related pro-environmental behavior as their own obligation, but there are more social and psychological obstacles for men to show as big willingness as women toward green purchasing behaviors. Despite the strong negative role that social gender stereotypes play in men’s eco-friendly consumption, Brough suggested that engagement of green consumption behaviors are also affected by the personal sensitivity about the maintenance of their gender identity. Individuals who are seriously concerned about their gender identity and are being more careful about their behavior that is associated with feminine characteristics may hold more intensive resist attitudes toward green consumption than men who care less about gender identity (Brough et al., 2016).

## Conclusion and Future Studies

In recent decades, byproducts of the explosive growth of the global economy, such as air and land pollution, forest degradation, reduction of freshwater, have caused a severe threat to the environment and led to a public health problem (Walker et al., 2014). Household material consumption accounts for almost 50% percent of total global material consumption, with a lot of them coming from food process and packaging manufacture industries. In addition to the energy consumption and overexploitation of natural resources, non-green consumption also leads to the severe contamination of the environment (Wang et al., 2014; Li et al., 2019). It is well-known that the carbon dioxide emission caused by driving significantly speeds up the greenhouse effect (Ivanova et al., 2016). Also, in the non-organic food planting, the usage of fertilizers and pesticides causes many emissions of greenhouse gas such as nitrous oxide and ammonia (Scheehle and Kruger, 2006; Webb et al., 2006), and the phosphorous from fertilizers leads to water eutrophication (Shenoy and Kalagudi, 2005), which damages the quality of water and causes the death of species living there. Considering the huge negative impact of manufacturing and retailing industries on the environment, researchers such as Tseng kept working on improving industrial sustainability from multiple perspectives such as technology and proposed many applicable suggestions to the current retailers and factories (Tseng et al., 2021). With the significant influence of gender on promoting public green consumption as we talked about earlier, this article made a comprehensive integration of the existing studies about the rationale behind gender different green consumption behavior, and therefore assisted the green marketing to design or promote green products following men’s and women’s preference respectively. Besides, since this article interpreted the VBN theory from the perspective of gender and analyzed how it affects gender different behavior on green consumption, future researchers can further explore what kind of factor that emerged from gender different beliefs and social norms can contribute to green consumption.

### Academic Contribution and Suggestions to Future Studies of Green Consumption

This article provides a comprehensive comparison of gender differences in green consumption behavior by integrating the previous findings and examining multiple reasons behind this difference from both personal and social aspects. Besides, by analyzing the men’s and women’s distinct green consumption from the VBN theory, we not only provided theoretical support for exploring individual differences in green consumption from the gender perspective but also found a unique insight to interpret the VBN theory. While an increasing number of scholars have started to pay attention to the gender differences in green consumption, current studies still have a lot of limitations and some aspects still need further exploration. First, this article revealed that gender differences in green consumption can be explained by the VBN theories, thus future studies can explore more about how to improve men’s green consumption from personal values aspects such as how to adjust men’s strong egoistic value to a more social-altruistic or biospheric value orientation, which then promotes their green consumption. Moreover, it is also critical to examine whether there are other contributing factors or any other ways to explain the gender differences. Since we found more influencing factors, more possible perspectives are available for us to explore the strategies of improving the green consumption of both men and women. In addition, as this article indicated, a large part of distinct results are generated in studies conducted in different countries, but for now, most data is still selected from developed regions. Thus, future researchers should increase the variety of the targeted population, that is, conducting studies in multiple regions especially those that are less-developed, and examine what kind of gender-related beliefs and norms exist in those regions.

### Green Marketing Implications

Among all the difficulties needed to be overcome in the process of accelerating worldwide green consumption, a crucial work is to increase men’s average green consumption intention and behavior, and to cultivate individuals’ ecological personal norm. Hence, it is important to strengthen the communication between academia and green marketing to prompt the publics’ green consumption based on the guidance of the VBN theory. This article reviewed gender-related beliefs and their linkage to green consumption, which provided some suggestions that might be helpful to green marketing. Based on previous researchers, there are several barriers to men’s green consumption, so following suggestions that intend to encourage men’s green consumption might be helpful for green marketers or policymakers. First, considering men’s worries about the effectiveness and practicality of green products, marketers must establish an explicit conversation with consumers to demonstrate the effectiveness of green products directly and therefore decrease men’s worries about the products’ effectiveness. Besides men’s concerns about the effectiveness of green products, their stronger egoism can also be used as an aspect to promoting green consumption. When advertising green products, instead of only focusing on their benefits to nature, they should also emphasize more about what kind of favors that green products can bring to men directly, such as saving energy or getting economic benefits. Another factor preventing men’s green consumption is that they tend to associate green products with femininity and show less preference about the products with green characteristics. Hence, setting a more masculine brand or product name is effective to attract men’s interest in green products and then increase their willingness to purchase these products. For example, a study in green business shows that after changing the traditional ecofriendly name of an electric car, BMW i3, to a more masculine phrase, “BMW Protection Model,” men become much more curious to know more about it (Brough et al., 2016). Therefore, future researchers can spend more energy on designing the brand name of eco-friendly products. Furthermore, because men usually worry about whether green consumption will affect their gender identity, exploring the direct or potential benefits that green consumption brings to men’s identity and publicizing these benefits in the community is also essential. For example, there is some research suggesting that men’s green consumption is one of the indicators of their reliability, and those who consume in an eco-friendly way tend to be favored and perceived as more reliable by women (Borau et al., 2020). Thus, we suggest that in the future, these related opinions should be presented as a public advertisement to reduce the impacts of the negative social stereotype of men’s environmental behavior on green consumption.

On the other side, to increase the frequency of women’s green consumption, emphasizing the benefits of green products to their family and the natural world, and improving women’s environmental knowledge are two unignorable points. Due to women’s higher altruism and their nurturing role in the family, it is effective to improve their green consumption by publicizing how green products directly or indirectly benefit their parents’ health and their surrounding environment. Moreover, as Lin suggested, individuals with a higher environmental knowledge are more likely to engage in green consumption (Lin and Niu, 2018). Therefore considering women’s relative weaker environmental knowledge (Diamantopoulos et al., 2003), government or academic institutions should pay attention to popularizing more environmental knowledge to women, which might improve and solidify their green consumption. Besides, in underdeveloped countries, the government should focus on taking action to improve both men’s and women’s environmental knowledge, such as popularizing environmental education in the whole society. Whereas, considering the environmental knowledge education is more generalized in the developed countries, promoting gender equality even further might be more helpful for these countries to increase the general public’s green consumption since it positively relates to the social engagement of green consumption (Li et al., 2019).

## Author Contributions

ZZ and YS: idea generation and validation. ZZ and YS: literacy searching. ZZ, YG, and YS: conceptualization. ZZ: writing—original draft preparation. ZZ, YG, YS, YL, and LZ: writing—review and editing. YS: funding acquisition and supervision.

## Conflict of Interest

The authors declare that the research was conducted in the absence of any commercial or financial relationships that could be construed as a potential conflict of interest.

## Publisher’s Note

All claims expressed in this article are solely those of the authors and do not necessarily represent those of their affiliated organizations, or those of the publisher, the editors and the reviewers. Any product that may be evaluated in this article, or claim that may be made by its manufacturer, is not guaranteed or endorsed by the publisher.
